# Ileal Adenocarcinoma with Liver Metastasis in Patient with Crohn's Disease: A 9-Year Survival

**DOI:** 10.1155/2019/8473829

**Published:** 2019-08-14

**Authors:** Jiten P. Kothadia, Deepa H. Nagaraju, Seymour Katz, Howard Bruckner, Steven H. Itzkowitz, Myron Schwartz

**Affiliations:** ^1^MUH James D. Eason Transplant Institute, University of Tennessee Health Sciences Center, 1265 Union Avenue, 4 Shorb Tower, Memphis, TN 38104, USA; ^2^UF Health Family Medicine and Pediatrics, 8274 Bayberry Road Jacksonville, FL 32256, USA; ^3^Department of Gastroenterology, NYU Langone Medical Center, 1000 Northern Blvd, Great Neck, NY 11021, USA; ^4^Bruckner Oncology, 2330 Eastchester Road, Bronx, NY 10469, USA; ^5^Division of Gastroenterology, Icahn School of Medicine at Mount Sinai, New York, NY 10029, USA; ^6^The Recanati/Miller Transplantation Institute, Icahn School of Medicine at Mount Sinai, New York, NY 10029, USA

## Abstract

Small bowel adenocarcinoma is a rare but well-known complication of Crohn's disease. The diagnosis of small bowel adenocarcinoma remains difficult since its presentation is highly variable and mimics active or obstructive Crohn's disease. The diagnosis is often delayed and typically detected only at surgery in an advanced stage with a poor prognosis. We report a case of metastatic ileal adenocarcinoma in a patient with Crohn's disease with prolonged survival. Our case describes serial promising treatment options of these advanced malignancies and raises a possible role for checkpoint immunotherapy.

## 1. Introduction

Small bowel adenocarcinoma (SBA) is a rare but well-known complication of Crohn's disease (CD) that was first described by Ginzburg et al. [1]. As per Surveillance, Epidemiology, and End Results (SEER) in 2018, it is expected that there will be 10,470 new cases of small bowel cancer [2]. The diagnosis of SBA remains difficult since its clinical presentation is highly variable and it may simulate active or obstructive CD [3, 4]. The diagnosis is often delayed and is typically detected at surgery in an advanced stage with a poor prognosis [5]. There is no clear guideline available for an optimal treatment regimen for either adjuvant chemotherapy or CD-associated metastatic SBA. The standard approach, emulating treatment regimens used for colorectal cancer, has had limited palliative success [6]. There is an essential need for the evaluation of novel strategies to treat these rare malignancies. We report a case of metastatic SBA in a patient with CD with prolonged survival.

## 2. Case Report

A 31-year-old Persian male with a family history positive for colon cancer in his grandfather had CD diagnosed since childhood. He underwent ileocolic resection for obstructive disease in 1996. He was maintained on mercaptopurine (6-MP) prophylaxis and did well until 2007 with occasional bouts of cramping and obstructive symptoms. He had a colonoscopy done in December 2007 that showed normal terminal ileum and colon. In February 2008, a computerized tomography (CT) scan of the abdomen showed two abnormal areas of stricture proximal and mid-ileum.

**Figure 1:**
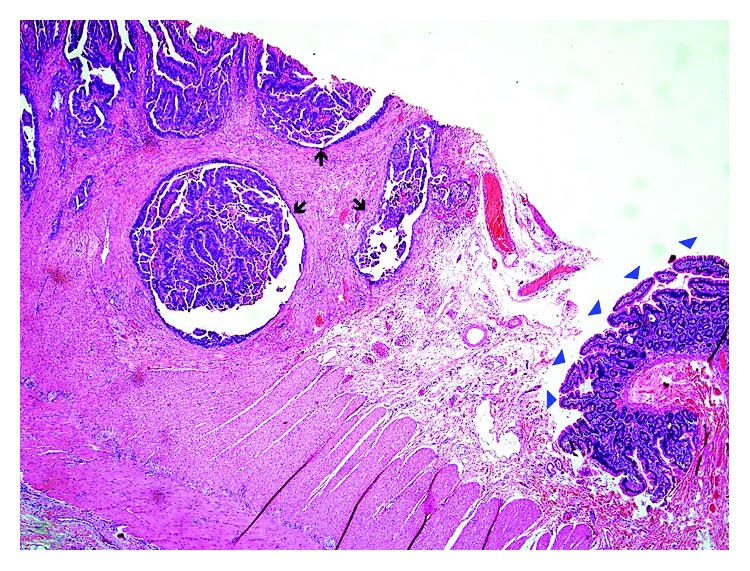
Histologic section from tumor mass shows normal small intestinal mucosa (blue arrowheads) and adjacent invasive adenocarcinoma (black arrows).

In June 2008, CT scan of the abdomen showed two areas of bowel dilation along with two hypodense liver lesions. In September 2008, the patient underwent resection of strictures and was found to have SBA as well as liver metastases. No lymph node (LN) dissection was performed because the diagnosis of cancer was not confirmed at the time of surgery. Histopathology showed poorly differentiated mucinous adenocarcinoma and underlying CD (Figure 1). The tumor infiltrated into the mesenteric fat and metastasized to the liver and adjacent LNs. It was classified as T4N1M1.

In October 2008, positron emission tomography (PET) scan showed nine metastatic liver lesions without other intra-abdominal uptake. He was given FOLFOX (leucovorin calcium, fluorouracil, and oxaliplatin) for a total of 12 cycles until chronic toxicity and weakness, due to poor nutrition, severe weight loss, poor fistula control, severe neuropathy, and moderate leucopenia and thrombocytopenia, limited further treatment (Table 1). A repeat PET scan, done in December 2008, showed interval resolution of the majority of the liver mets except 2 enhancing lesions on the left liver lobe and the stable appearance of small bowel thickening. He underwent left liver lobectomy (segment 7) for the metastatic tumor in January 2009. He then had four additional cycles of FOLFOX. FOLFOX was discontinued when he developed severe weight loss, worsening severe neuropathy, and chronic moderate granulocytopenia and thrombocytopenia resulting discontinuation of standard chemotherapy. In December 2011, imaging patient was noted to have enlarged LNs in the small bowel mesentery confirming metastatic SBA. In April 2012, he was hospitalized with abdominal cramping, distention, and hematochezia in the setting of metastatic SBA. He underwent resection of the large mesenteric mass of matted LNs resulting stricture proximal to the previous ileocolic anastomosis and below a prior jejunojejunostomy.

The patient was considered to be refractory due to widespread disease progression to the peritoneal LNs, liver, and lung, and his condition was unfit for standard treatments due to severe weight loss, fistulae, neuropathy, and chronic poor marrow reserve. Treatment began with half the standard dosage of all drugs (FOLFOX) with added gemcitabine and as tolerated irinotecan (GFLIO) and nutritional support. Follow-up positron emission tomography (PET) scan in May 2015 showed no hypermetabolic activity with normal tumor markers. During this time, the patient continued with a fistulizing CD with infection, obstruction, and dehydration requiring hospitalization and mesenchymal stromal cell (MSC) infusion in December 2013 for the fistulizing CD. CT scan in May 2014 and September 2015 revealed stable disease without evidence of recurrence. PET scan done in November 2015 showed the progression of the tumor.

Further treatment that began with Cetuximab along with gemcitabine, 5-fluorouracil, irinotecan, leucovorin, and oxaliplatin (GFLIO) produced a period of relatively stable disease with improved tumor markers, improved area of stable disease by RECIST criteria.

In May 2016, surveillance imaging revealed the patient had increased pulmonary nodules and metastasis to the thoracic spine (T6-7). The patient underwent T6-7 laminectomy and resection of the extradural tumor in February 2017 due to the threat posed by symptomatic bony metastasis. Trastuzumab was added as the tumor was human epidermal growth factor receptor 2-positive (HER-2/neu amplified FISH 2.42). This produced regression of pulmonary metastatic disease and a fall in CEA. The bony disease became stable and was technically unevaluable. In March 2018, the patient was admitted to the hospital again with gastrointestinal (GI) bleeding from a Crohn's related enterocutaneous fistula. He was found to have a recurrence of tumor at the enterocutaneous fistula site with GI bleeding from peristomal varices due to portal hypertension. His condition was deemed nonsurgical. The patient was hospitalized again in June 2018 and unfortunately succumbed to metastatic disease. A detailed treatment course with the outcome is summarized in Table 1.

## 3. Discussion

Small bowel adenocarcinoma (SBA) is a rare but well-known complication of CD [7]. The cumulative risk of SBA in CD is reported as 2.2% in 25 years of CD [4]. Bojesen et al. [8] showed a standardized incidence ratio of developing SBA in patients with CD to be 14.38 (95% confidence interval 8.78-22.20) compared to the general population. The study showed the absolute risk of SBA in patients with CD to be 9 per 100,000/year. In addition, the majority of the patients (57%) with CD-associated SBA had moderate to severe disease with small bowel and upper gastrointestinal tract involvement [8]. The diagnosis of SBA remains challenging due to the nonspecific presentation which can mimic active or obstructive CD [3, 4]. Imaging studies may be confused with other complications of CD such as an abscess, a stricture, or an inflammatory mass [4]. There are no specific guidelines for SBA screening in CD patients, and thus, it is typically detected at an advanced stage with poor prognosis [3, 7]. At best, a two-year survival rate for CD-associated SBA was 27% [9].

The treatment options for CD-associated metastatic SBA are limited, and the evidence for the routine use of adjuvant chemotherapy is still lacking. The general approach has been to imitate the treatment regimens used for colorectal cancer. There is evidence of beneficial effects regarding response and survival using 5-FU in combination with platinum agents and retrospective studies describing the use of irinotecan ±5-FU [10, 11]. The role of active targeted therapies with antiepidermal growth factor receptor (EGFR) and vascular endothelial growth factor (VEGF) receptor therapies has not been established [11]. Tsang et al. described a case of advanced SBA treated with bevacizumab with gemcitabine and oxaliplatin [12]. Research with basket protocols suggests a general role for Trastuzumab as a treatment for HER2 positive GI cancers [13].

There is no clear guideline for an optimal treatment regimen with primary chemotherapy for patients with advanced SBA. Prior retrospective studies reported no significant survival benefit for patients who received primary chemotherapy after resection of their primary tumors [5, 14, 15]. However, a retrospective study demonstrated that the use of adjuvant chemotherapy for patients with resected SBA was associated with increased disease-free survival [14]. The feasibility and efficacy of gemcitabine, 5-fluorouracil, irinotecan, leucovorin, and oxaliplatin (GFLIO) have been demonstrated in patients with metastatic pancreatic cancer. The rationale for the use of GFLIO in the patient population was designed to approximate sequence-dependent synergistic effects while minimizing the sequence-dependent toxic effects among the four drugs. The GFLIO regimen allows for the simultaneous administration of four drugs, safely, at half their standard dosages. These drugs produce six synergistic drug pairs. The combination and several of the drug pairs have reversed drug resistance of many gastrointestinal, gynecological, and genitourinary adenocarcinoma. The regimen was thought to reverse or delay the resistance of standard treatment when used in combination [16]. Schrock et al. studied the large-scale genomic profiling of SBA and comparison with colorectal cancer (CRC) and gastric carcinoma (GC) [17]. The distribution of biomarkers favors further testing of both gemcitabine and irinotecan [18].

There is an essential need for novel strategies to treat these rare malignancies. Salem et al. [18] compared molecular variations between SBA, right-sided colon cancers, and gastroesophageal cancers and showed that frequently mutated genes in SBA were *TP53* (*51%*), *KRAS* (*49%*), *APC*, *SMAD4*, *PIK3CA*, *BRAF*, *CTNNB1*, *ATM*, *ERBB2*, and *BRCA2*. The study demonstrated that though SBA has some molecular features in common with CRC and GC, it indeed represents a unique genomic entity. In addition, *immunohistochemistry* (*IHC*) evaluation of ~531 SBA patients showed several biomarkers that include low RRM1 (80%), high TOPO1 (41%), low TUBB3 (64%), low ERCC1 (76%), and low TS (59%) had a favorable role for chemotherapy drugs with gemcitabine, irinotecan, taxanes, oxaliplatin, and fluorouracil (5-FU), respectively [18]. In our patient, GFLIO chemotherapy was started prior to obtaining these proteomic markers.

## 4. Conclusion

We report a case of metastatic SBA in a patient with CD with prolonged survival of more than nine years on treatment with chemotherapy. Our case of prolonged survival and review of the GFLIO experience also found a ten-year disease-free survivor following “adjuvant” or what had been intended as palliative GFLIO therapy. This patient initially had residual lymph node and margin positive SBCA [10]. These few long survivors suggest a possible role for checkpoint immunotherapy. In addition, immunotherapy in expert hands is no longer contraindicated for CD. Also, when there is a rare neoplastic disease with long survivorship, the chemical profile of these survivors warrants identification. The feasibility of (further) treatment of fragile and resistant patients (with moderate dosages of combination chemotherapy and guidance provided by biomarker assays) is noteworthy.

## Figures and Tables

**Table 1:** Treatment summary and clinical outcome.

Date	Treatment (medical, surgical)	CEA (ng/ml) and CA 19-9 (U/ml) range during treatment period	Outcome	Complications
1996	Ileocolic resection for obstructive disease, maintained on 6-MP		Tolerated procedure well	(i) Was well until June 2008, when noted to have two areas of small bowel dilation along with two hypodense liver lesions
September 2008	Underwent resection of strictures was found to have SBA	CEA: 0.1-0.9	Tolerated procedure well	(i) PET scan showed nine metastatic liver lesions
October 2008	Chemotherapy with FOLFOX (leucovorin calcium, fluorouracil, and oxaliplatin) for 12 cycles	CEA: 0.1-0.9	PET scan in December 2008 showed interval resolution of majority of the liver mets except 2 enhancing lesions on the left liver lobe with the stable appearance of small bowel thickening	(i) Weight loss, fistulae, failure to thrive, and bone marrow suppression
January 2009	Left hepatic lobe resection for isolated liver metastasis Received 4 additional cycles of chemotherapy with FOLFOX		Tolerated procedure well	(i) Patient had treatment response until December 2011 when noted to have widespread disease progression to the peritoneal LNs, liver, and lung (ii) In April 2012, he was hospitalized with obstructive symptoms. He underwent resection of the large mesenteric mass of matted LNs resulting stricture
April 2012-May 2015	Gemcitabine, 5-flourouracil, irinotecan, leucovorin, and oxaliplatin (GFLIO)	CEA: 0.1 to 2.7 CA 19-9: 13 to 32	Stable disease without evidence of recurrence on PET scan in May 2015 and September 2015	(i) Continued fistulizing CD requiring mesenchymal stromal cell (MSC) infusion in December 2013 (ii) PET scan in November 2015 showed progression of the tumor
November 2015-May 2016	Cetuximab + (GFLIO)	CEA: 0.3 to 2.9 CA 19-9: 28 to 71 *After May 2016*: CEA: 0.3 to 21 CA 19-9: 91 to 567	Clinically stable without progression	(i) May 2016: disease metastasized to the lungs and thoracic spine requiring laminectomy and resection of the extradural tumor and radiotherapy to spinal metastasis in March 2017
July 2017–February 2018	Trastuzumab was added for HER2+ with Cetuximab + (GFLIO)	CEA: 9.5 to 41 CA 19-9: 398 to 1338	Clinically stable	(i) Patient remained clinically stable
March 2018				(i) Recurrence of tumor at fistula site with bleeding
June 2018				(i) Died due to complication from metastatic SBA

∗∗∗6-MP: mercaptopurine; CEA: carcinoembryonic antigen; CA 19-9: cancer antigen 19-9; CD: Crohn's disease; HER2+: human epidermal growth factor receptor 2; PET: positron emission tomography.
